# Generative AI Decision-Making Attributes in Complex Health Services: A Rapid Review

**DOI:** 10.7759/cureus.78257

**Published:** 2025-01-30

**Authors:** Nandini Doreswamy, Louise Horstmanshof

**Affiliations:** 1 Faculty of Health Sciences, Southern Cross University, Lismore, AUS; 2 Health Sciences, National Coalition of Independent Scholars, Canberra, AUS

**Keywords:** artificial intelligence, decision making, gen ai attributes, gen ai decision making, generative artificial intelligence, healthcare, health policy, health regulation

## Abstract

The advent of Generative Artificial Intelligence (Generative AI or GAI) marks a significant inflection point in AI development. Long viewed as the epitome of reasoning and logic, Generative AI incorporates programming rules that are normative. However, it also has a descriptive component based on its programmers’ subjective preferences and any discrepancies in the underlying data. Generative AI generates both truth and falsehood, supports both ethical and unethical decisions, and is neither transparent nor accountable. These factors pose clear risks to optimal decision-making in complex health services such as health policy and health regulation. It is important to examine how Generative AI makes decisions both from a rational, normative perspective and from a descriptive point of view to ensure an ethical approach to Generative AI design, engineering, and use.

The objective is to provide a rapid review that identifies and maps attributes reported in the literature that influence Generative AI decision-making in complex health services. This review provides a clear, reproducible methodology that is reported in accordance with a recognised framework and Preferred Reporting Items for Systematic reviews and Meta-Analyses (PRISMA) 2020 standards adapted for a rapid review. Inclusion and exclusion criteria were developed, and a database search was undertaken within four search systems: ProQuest, Scopus, Web of Science, and Google Scholar.

The results include articles published in 2023 and early 2024. A total of 1,550 articles were identified. After removing duplicates, 1,532 articles remained. Of these, 1,511 articles were excluded based on the selection criteria and a total of 21 articles were selected for analysis. Learning, understanding, and bias were the most frequently mentioned Generative AI attributes.

Generative AI brings the promise of advanced automation, but carries significant risk. Learning and pattern recognition are helpful, but the lack of a moral compass, empathy, consideration for privacy, and a propensity for bias and hallucination are detrimental to good decision-making. The results suggest that there is, perhaps, more work to be done before Generative AI can be applied to complex health services.

## Introduction and background

The concept of artificial intelligence (AI) can be traced back to the 1940s, when science fiction writer Isaac Asimov wrote Runaround, a famous story about a robot [1]. Haenlein and Kaplan [2] define AI as “a system’s ability to interpret external data correctly, to learn from such data, and to use those learnings to achieve specific goals and tasks through flexible adaptation”. There are many types of AI, and it is a challenge to classify them. Industry reports tend to classify AI according to its applications. This includes applications in cognitive science, such as learning systems and neural networks; robotics applications, such as visually perceptive AI and navigation-related AI; and natural interface applications, such as natural language processing and virtual reality.

In 1950, Turing published Computing Machinery and Intelligence, a seminal article on intelligent machines, in which he offered a way to test machine intelligence [3]. He postulated that if a human interacts with a machine and is unable to distinguish the machine from another human being, then the machine should be considered intelligent. This test, called the Turing test, remains a touchstone for AI [3].

Weizenbaum [4] claimed that ELIZA, a computer system he invented, passed the Turing test. However, this is debatable, as Turing did not specify the sophistication or skill of the human investigator who interacts with the machine [5]. More recently, the AI known as Generative Pre-trained Transformer (GPT) is able to write articles as sophisticated as the works of a human writer [6]. As such, it may have passed the Turing test [3].

GPT is arguably the most advanced AI in the world today. It is the engine that powers popular AI applications, such as ChatGPT. Developed by the American research organisation OpenAI, with significant funding from Microsoft Corporation, ChatGPT is a large language model (LLM) with over 1.6 trillion parameters. It is capable of passing difficult exams, writing convincing essays, and chatting so fluently that many human beings cannot distinguish it from another human. However, as of July 2023, ChatGPT still fails a crucial test: it cannot solve simple puzzles that require visual logic [7].

Types of AI

**Figure 1:**
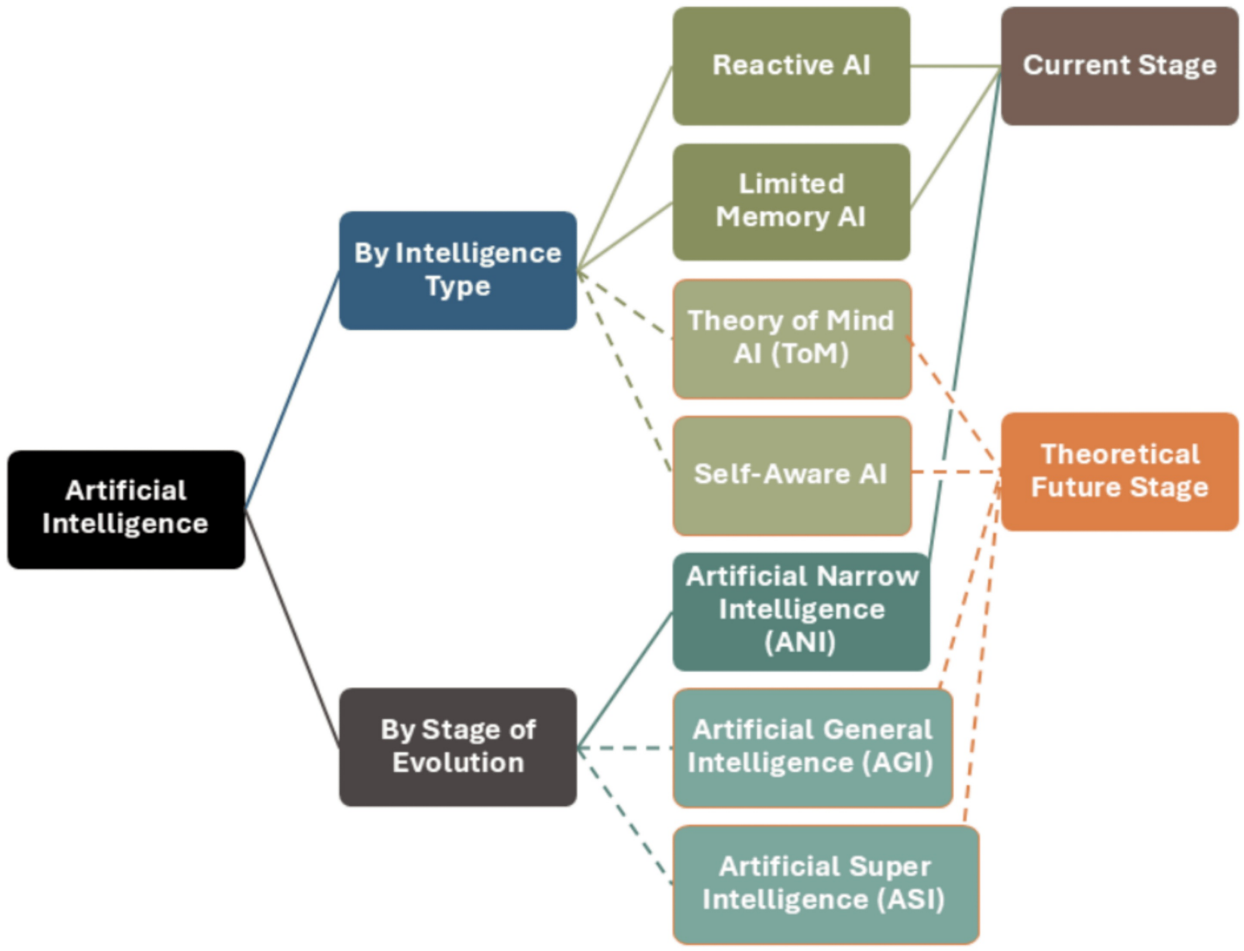
Classification and relationships between types of AI. Note: This image is the authors' own creation.

AI can be divided into cognitive, social, and emotional AI, based on the type of intelligence that is harnessed. AI may also be classified by function, for instance, Interactive AI, Analytic AI, Text AI, and Visual AI. Viewed through the lens of decision-making, however, it is important to classify AI in relation to its similarity to the human mind. From the decision-making perspective, AI can be divided into Reactive AI, Limited Memory AI, Theory of Mind AI (ToM), and Self-aware AI [8,9]. Research into the Theory of Mind AI (ToM) has been foundational in understanding AI's decision-making capabilities [10]. Figure 1 shows the classification of AI and the relationships between various types of AI.

Reactive AI reacts to inputs by producing predictable outputs, based on its programming [11]. It does not form memories or use experience to make decisions. This type of AI is incapable of using past decisions, right or wrong, to inform decisions in the future [12]. Machine learning (ML) models are usually based on Reactive AI [13]. Examples are Deep Blue--IBM's AI that plays chess [12], and entertainment giant Netflix's recommendation engine [14].

Limited Memory AI can make decisions based on learning from "past" data [15]. While it can look into the past, recognise patterns and changes, and monitor objects or events over a period of time, its memory is short-lived [15,16]. Self-driving cars are an example of this type of AI [16].

Theory of Mind refers to the human meta-representational ability--the ability to ascribe mental states to oneself and to others and conceptualise and reason about mental states [17]. Theory of Mind is called a "theory" because it predicts the mental state of other entities or organisms, which cannot be directly observed, and predicts their behaviour [18,19]. It involves two levels of social cognition: fast, automatic processes and slow, conscious, rational processes [20,21]. Theory of Mind is a fundamental component of social interaction--when applied to AI, it provides the ability to form an idea about the mental states of other entities, including humans.

Self-aware AI is also theoretical at the present time. The concept is an AI with effective adaptive skills on par with humans, capable of organising the results of conscious processing and using human-like awareness to make conscious decisions in everyday living [22]. Self-aware AI will also understand its environment, learn from experience, demonstrate that it knows that it has learned, and understand how it has learned [23].

Based on the stage of evolution, AI can also be classified into Artificial Narrow Intelligence (ANI), Artificial General Intelligence (AGI), and Artificial Super Intelligence (ASI) [2]. ANI has a narrow set of human-like capabilities and can independently perform a small range of tasks with these capabilities. Machine learning systems, deep learning systems, and Generative Artificial Intelligence (Generative AI or GAI) are considered to be ANI. On the other hand, AGI is equal to human intelligence in its multi-dimensional capability, including learning, perception, and the ability to form complex connections. Finally, ASI is said to be capable of outperforming humans in every aspect of human ability because it has more memory, enhanced abilities in data processing and analysis, and superior decision-making skills [2].

Generative AI

With the advent of Generative AI, artificial intelligence is making quantum leaps in its evolution and maturity [24]. Generative AI consists of models and algorithms that can generate new and unique content [24]. Claude AI, ChatGPT, Google Bard, and Perplexity AI are among the leading Generative AI today, in terms of their performance in complex scenarios, including medical decision-making [25]. The organisations that created and continue to develop these Generative AI are Anthropic Public-Benefit Corporation (Anthropic PBC), Google Limited Liability Company (Google LLC), Open Artificial Intelligence Technologies, Incorporated (OpenAI), and Perplexity AI, Incorporated (Perplexity AI, Inc.), respectively, all based in the United States. Other Generative AI applications include DALL-E and Midjourney, which can create photo-real images and futuristic architecture [26]; Murf AI, which can generate synthetic voices or replicate voices [27]; and AlphaCode, which can generate programming code at the level of a professional programmer [28].

Generative AI has the ability to make complex and nuanced decisions, allowing it to take on creative and innovative roles that once required human versatility. However, with Generative AI, there is a risk of generating inaccurate, incorrect, or irrelevant results. The usual reason to query a Generative AI like ChatGPT is to obtain an answer that is an abstract, not an extract, of data [29,30]. Human evaluation of the factual accuracy of these answers shows that Generative AI like ChatGPT can provide fabricated answers that may be partially or completely false [31]. This calls the reliability of Generative AI into question and poses considerable risk. For instance, AI hallucinations can result in incorrect diagnoses or treatment plans that harm patients in the real world [32].

AI hallucinations may be intrinsic, where the output generated contradicts the source data, or extrinsic, where the output cannot be verified based on the source data, as the data neither supports nor contradicts it [33]. Studies have shown that intrinsic and extrinsic AI hallucinations can occur through distinct mechanisms and require different mitigation strategies [34]. In a process known as semantic drift [30], the AI's answer drifts away from accuracy, which can also be considered as one of the definitions of AI hallucination. AI hallucinations may be one of the most significant problems relating to Generative AI [31]--so significant, in fact, that Rawte et al. [35] introduced the Hallucination Vulnerability Index (HVI)--a framework that quantifies the spectrum of hallucinations, and evaluates and ranks LLMs by their vulnerability to this phenomenon.

Gao et al. [36] tested AI hallucinations in an experiment where they prompted ChatGPT to generate abstracts based on fifty articles across five journals. They found that the AI fabricated a number of abstracts from data it had generated, in a way that made it difficult to determine whether an abstract was written by the LLM or a human being. ChatGPT’s ability to fabricate data, the ambiguity around its authorship, and the resulting lack of accountability, are all matters of concern. Dziri et al. [37] found that hallucinations are caused by insufficient, incorrect, or biased training data. AI models seem to amplify these flaws. Alkaissi and McFarlane [38] queried ChatGPT itself on AI hallucinations; the answer was, “…there have been instances where advanced AI systems, such as generative models, have been found to produce hallucinations, particularly when trained on large amounts of unsupervised data”.

The European Association for Viewers Interests (EAVI), a media literacy organisation established by the European Commission, states that the process by which AI generates responses remains opaque, with users merely receiving and consuming the answers that it produces [39]. Are hallucinations, then, a result of programming errors and flawed AI algorithms? Or is it possible that ChatGPT, and AIs like it, will approach--or are approaching--Artificial General Intelligence, with a human-like ability to tell the truth some of the time, and lie, occasionally?

Based on what is known about the process by which Generative AI generates answers, it may not, in fact, be deceptive in a human way. Rather, it may be generating incorrect or irrelevant responses in a manner similar to biological cells generating faulty proteins because of transcription errors. The biological process of transcription is based on deoxyribonucleic acid (DNA)--the stable, storage form of genetic data--which is converted into ribonucleic acid (RNA), when needed. RNA provides the blueprint for the synthesis of required proteins. Similarly, the process of prompting a Generative AI is based on the stored data associated with the AI, which it converts into responses. Similar to the transcription of DNA to RNA, a Generative AI, in the process of generating a response to a prompt, converts data from a storage form to a functional, usable form that fulfils a requirement.

The margin of error in these processes can also be compared. Cellular processes are very precise, with complex safety mechanisms in place. Nevertheless, occasional errors in transcription do occur that result in disease [40]. Similarly, as Generative AI develops further, accuracy may well increase, but eventually, AI hallucinations and their resulting harms may be unavoidable.

McIntosh et al. [41] undertook a survey to explore the Generative AI landscape. They found that the next generation of AI, such as Q* (Q-star) from OpenAI, will integrate LLMs like ChatGPT, reinforcement learning algorithms like Q-learning, and pathfinding AI like A* (A-star). This will enable an AI that is adept at communication, reasoning, and structured tasks. capable of learning from its interactions and optimising its decision paths. Therefore, AIs like Q-star will become more adaptable and intelligent over time [41]. If they are not at the level of AGI now, where they meet or surpass human intelligence, it may be an inevitable milestone in AI development in the not-too-distant future.

Advances in AI decision-making

Advances in AI decision-making reflect an acceleration in its development, beyond algorithms, data-based inputs, and machine learning. Although it is unlikely that AI has evolved into Artificial General Intelligence (AGI), advanced AI is approaching this level of evolution. For instance, fuzzy logic, or multi-valued logic [42], can now be incorporated into AI systems. This has applications in reasoning and decision support [43], where it can improve decision-making under uncertainty.

AI’s increasing sophistication in problem-solving and complex computation may even make it essential to the development of society itself [44]. There is a pressing need to ensure that AI makes decisions that are transparent, explainable, equitable, and responsible. This need has resulted in a push towards eXplainable AI (XAI) and Responsible AI (RAI) [45]. These types of AI have the ability to explain their reasoning, strengths and shortcomings, and future behaviours.

AI in healthcare

Wilson and Daugherty [46] postulate that humans and AI are in the process of merging to form a collaborative intelligence. The authors argue that humans must assist AI to ensure optimal outcomes. They must train the AI in a task-appropriate manner, explain the outcomes of these tasks, and ensure that AI is used responsibly, in a manner that benefits human beings and prevents harm [46]. However, in healthcare, the social contract, economic and political factors, and contemporary agreed norms determine the nature and role of AI in healthcare [47]. Most healthcare organisations lack the investment and infrastructure to collect the data required to train AI appropriately, identify local patterns, and focus on the values and needs of the local population. They also lack the expertise to identify and weed out bias arising from the use of AI. Jandrić [48] states that AI can not only echo bias already found in data, but can also reinforce prejudicial attitudes. Furthermore, it can recombine existing prejudices to generate new biases. Iqbal et al. [49] emphasize the importance of considering and incorporating ethical principles alongside AI capabilities to ensure the impartiality of AI in healthcare.

Complex health services are beginning to incorporate several advanced AI techniques, such as deep learning and natural language processing [50], into evidence-based and probability-based AI decision systems [51]. At the present time, data are being generated at a rate that exceeds the human cognitive capacity to manage it [52]. Therefore, while humans currently dominate decision-making in complex health services, advanced AI, such as Generative AI, has ever-increasing utility and influence. It seems only a matter of time before Generative AI begins to drive or dominate decision-making in complex health services. Therefore, it is timely and essential to explore the following research question: What attributes have been reported in the literature that influence Generative AI decision-making in complex health services?

Rationale and objectives

Generative AI is viewed as the epitome of reasoning and logic based on clearly defined inputs. However, while the rules and inherent logic in Generative AI programming are normative, there may be a descriptive component based on its programmers’ subjective preferences, beliefs, or bias, and based on errors and bias in the data used to train Generative AI. As the role of Generative AI expands across complex health services such as health policy and health regulation, it is important to identify and analyse the attributes that influence Generative AI decision-making, not only from a rational, normative perspective, but also from a descriptive point of view.

Healthcare systems are increasingly adopting Generative AI for complex decision-making. Therefore, healthcare leaders and policymakers require a comprehensive understanding of Generative AI attributes that influence its decision-making, in order to develop appropriate governance frameworks and guidelines for its regulation and use [53]. Identifying and analysing these attributes also helps in anticipating and mitigating potential risks associated with Generative AI deployment in complex healthcare settings [54]. Therefore, this rapid review was undertaken to identify and map attributes reported in the literature that influence decision-making by Generative AI in complex health services.

Review question

The review was designed to answer the following research question:

What attributes have been reported in the literature that influence AI decision-making in complex health services?

## Review

Methods

The review provides a clear, reproducible methodology and is reported in accordance with Preferred Reporting Items for Systematic reviews and Meta-Analyses (PRISMA) 2020 standards, adapted for a rapid review [55].

Framework

This review is reported in accordance with the framework recommended by Dobbins [56]. The review focuses on a topic that is not only emerging, but also evolving rapidly. Therefore, a rapid but rigorous review of the literature was appropriate here.

Search Strategy

An initial informal exploration was undertaken to determine the optimal search system and database combinations. Suitable search systems were identified--ProQuest, Scopus, Web of Science, and Google Scholar (GS). Search terms and a search strategy were defined for each of these systems (Table 1).

**Table 1:** Search systems, databases, and search terms used to identify literature for review. Search was conducted on 11 February 2024.

Search system	Search terms	Number of articles
ProQuest (All ProQuest databases)	Search terms: abstract(Generative Artificial Intelligence) OR abstract(Gen AI) OR abstract(ChatGPT) OR abstract(DALL-E) OR abstract(GPT) AND abstract(decision making) OR abstract(decision) AND abstract(factors) OR abstract(attributes) AND abstract(health policy) OR abstract(healthcare) OR abstract(health regulation)	1,176
	Limiters: Limited to peer reviewed articles published in English in 2023 and 2024, where the full text was available.	
	Subjects excluded from the search results: patients AND covid-19 AND medical personnel AND pandemics AND hospitals AND questionnaires AND mortality AND coronaviruses AND mental health AND infections AND nurses AND diabetes AND quality of life AND primary care AND womens health AND pediatrics AND intervention AND medical research AND interviews AND emergency medical care AND older people AND professionals AND education AND age AND mental disorders AND cancer AND physicians AND severe acute respiratory syndrome coronavirus 2 AND chronic illnesses AND communication AND telemedicine AND clinical trials AND disease AND disease transmission AND cross-sectional studies AND surgery AND sustainability AND clinical outcomes AND covid-19 vaccines AND clinical medicine AND caregivers AND medical diagnosis AND antibiotics AND pregnancy AND immunization AND comorbidity AND epidemiology AND medical prognosis AND vaccines AND cancer therapies AND hospitalization AND cardiovascular disease AND mental depression AND disease prevention AND children AND hypertension AND students AND gender AND patient safety AND classification AND anxiety AND climate change AND medicine AND proteins AND energy consumption AND sustainable development AND consumers AND innovations AND case studies AND supply chains AND land use AND manufacturing AND developing countries--ldcs AND food AND marketing AND blockchain AND agriculture AND infrastructure AND metabolism AND households AND agricultural production AND cryptography AND emissions AND medical imaging AND physiology AND carbon AND metabolites AND employment AND urban areas AND data mining AND databases AND geographic information systems AND environmental impact	
	Subjects included in the search results: health care OR decision making OR health services OR public health OR artificial intelligence OR data collection OR algorithms OR qualitative research OR machine learning OR risk factors OR statistical analysis OR regression analysis OR systematic review OR literature reviews OR research OR collaboration OR health care policy OR datasets OR software OR variables OR accuracy OR data analysis OR neural networks OR health risks OR population OR health care industry OR sociodemographics OR health facilities OR social networks OR deep learning OR attitudes OR knowledge OR trends OR ethics OR electronic health records OR socioeconomic factors) AND (sensors OR optimization OR methods OR chatbots OR decision analysis OR risk assessment OR automation OR efficiency OR decision trees OR internet of things OR mathematical models OR privacy OR language OR design OR research methodology OR behavior OR simulation OR hypotheses OR support vector machines OR remote sensing OR performance evaluation OR influence OR artificial neural networks OR research & development--r&d OR productivity OR big data OR learning algorithms OR mathematical analysis OR prediction models OR time series OR technology OR economic development OR multiple criterion OR fuzzy sets OR evaluation OR analytic hierarchy process OR economic growth OR generative artificial intelligence OR natural language processing	
Web of Science (All Web of Science databases)	Search terms: (((((((((((TI=(Generative Artificial Intelligence)) OR TI=(Gen AI)) OR TI=(ChatGPT)) OR TI=(DALL-E)) OR TI=(GPT)) AND TI=(decision making)) OR TI=(decision)) AND TI=(factors)) OR TI=(attributes)) AND TI=(health policy)) OR TI=(healthcare)) OR TI=(health regulation)	60
	Expansion of abbreviations: TI=Title	
	Limiters: Limited to the category of ‘highly cited papers’ and ‘hot papers’ within the search results, and limited to peer reviewed articles published in English in 2023 and 2024. The Web of Science (Clarivate) LibGuide defines ‘highly cited papers’ and ‘hot papers’ as follows: ‘Highly cited papers’ are papers that perform in the top 1% based on the number of citations received when compared to other papers published in the same field in the same year. ‘Hot papers’ are papers published in the last two years that are receiving citations quickly after publication. These papers have been cited enough times in the most recent bimonthly period to place them in the top 0.1% when compared to papers in the same field and added to the database in the same period.	
Scopus (The Scopus database)	Search terms: TITLE-ABS-KEY ( Generative AND Artificial AND Intelligence ) OR TITLE-ABS-KEY ( Gen AND AI ) OR TITLE-ABS-KEY ( ChatGPT ) OR TITLE-ABS-KEY ( DALL-E ) OR TITLE-ABS-KEY ( GPT ) AND TITLE-ABS-KEY ( decision AND making ) OR TITLE-ABS-KEY ( decision ) AND TITLE-ABS-KEY ( factors ) OR TITLE-ABS-KEY ( attributes ) AND TITLE-ABS-KEY ( health AND policy ) OR TITLE-ABS-KEY ( healthcare ) OR TITLE-ABS-KEY ( health AND regulation )	14
	Expansion of abbreviations: TITLE-ABS-KEY = Title, Abstract, or Keyword	
	Limiters: Limited to peer reviewed articles in English, published in 2023 and 2024.	
	Note: The fact sheet about Scopus states that the Scopus database consists of peer reviewed literature only.	
Google Scholar	Search terms: (Generative Artificial Intelligence) OR (Gen AI) OR (ChatGPT) OR (DALL-E) OR (GPT) AND (decision making) OR (decision) AND (factors) OR (attributes) AND (health policy) OR (healthcare) OR (health regulation)	300
	Limiters: Limited to articles in English published in 2023 and 2024. Of the 5,180 results, only the first 300 were considered [57].	
Total records identified by the database search		1,550
Total records after duplicates were removed		1,532

All available databases in these systems were searched for relevant literature. The most recent search was undertaken on 11 February 2024. As recommended by Haddaway et al. [57], only the first 300 results were considered on Google Scholar (GS).

GS displays search results based on a number of parameters, including the recent popularity of articles displayed [58]. This may lead to an issue with the replicability of search results [59]. Nevertheless, GS is a useful supplement to other search methods [60].

This review considers a wide range of evidence sources, including empirical research (qualitative and quantitative studies), case studies, expert opinions, critiques, commentaries, editorials, textual data, and narrative data. Search terms were developed that are broad enough in scope to include all relevant literature. The search terms are as logical, relevant, and comprehensive as possible.

The search was limited to articles published in English in 2023 and 2024 only as the research question pertains to an emerging topic. Article screening and selection were based on the updated guidelines for reporting systematic reviews (PRISMA 2020) [55].

Inclusion and Exclusion Criteria

Detailed inclusion and exclusion criteria are listed in Table 2.

**Table 2:** Inclusion and exclusion criteria.

Inclusion criteria	Exclusion criteria
Article type	
Peer-reviewed articles	All other article types
Language	
English	All other languages
Year of publication	
2023 and 2024	Articles before 2023
Other criteria	
Articles were included if they related to: Generative AI; and attributes of Generative AI; and complex health services, i.e., non-clinical healthcare such as health policy, health regulation, public health, and population health.	Articles on topics not relevant to the research question were excluded-topics such as: clinical health, clinical decision support systems, decision space for health recruitment, legal matters, forms or types of AI other than Generative AI environmental health, contamination, and toxicity, mathematical modelling, AI algorithms, and assessment of organisational performance.

Data Extraction, Appraisal of Study Quality, and Validity of Results

Duplicates were removed from the search results. The first author reviewed the titles and abstracts of the remaining articles and applied inclusion and exclusion criteria. The second author reviewed this. Both authors then evaluated the full text of the remaining articles. Both authors cross-checked the extracted data to minimise personal bias [61]. Any disagreements on data extraction were resolved through detailed discussions until a consensus was reached.

Data Analysis

A thematic analysis was undertaken in order to identify the attributes of Generative AI mentioned in the articles included, count the frequency of these attributes, and present the results in tabular and diagrammatic form.

Results

**Figure 2:**
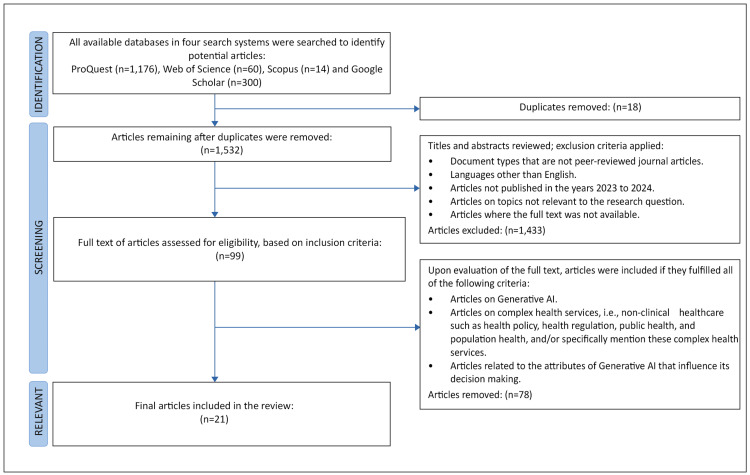
Flow diagram adapted from the PRISMA 2020 guideline for reporting systematic reviews. Adapted from Ref. [55]. PRISMA: Preferred Reporting Items for Systematic Reviews and Meta-Analyses.

A total of 1,550 articles were identified, and 18 duplicates were removed. The titles and abstracts of the remaining 1,532 articles were screened against inclusion and exclusion criteria, with 1,433 articles excluded. Both authors read the full text of the remaining 99 articles, excluding 78 of these articles, as they did not relate to Generative AI, the attributes of Generative AI, or complex health services such as health policy and health regulation. The remaining 21 articles were deemed relevant to the research question. Figure 2 shows the adapted PRISMA flow diagram of article screening and selection [55]. The results are presented below.

Categories and Frequency of Attributes

**Figure 3:**
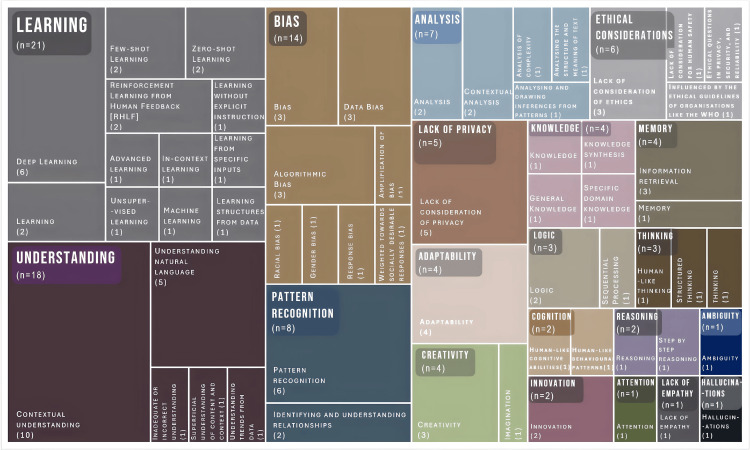
Attributes of Generative AI that influence its decision-making in complex health services and frequency of attributes (n=111) mentioned in the articles included. Note: This image is the authors' own creation.

In the articles included, 20 categories of attributes were identified, with 111 sub-categories. Learning is mentioned 21 times--it is the most frequent attribute. This is followed by understanding, which was mentioned 18 times. Bias is mentioned 14 times. Pattern recognition is mentioned eight times; analysis, seven times; ethical considerations, six times; lack of privacy, five times; and knowledge, memory, adaptability, and creativity, four times each. The frequency of attribute categories and sub-categories are visualised in Figure 3.

Three broad types of attributes were identified. A total of 13 attributes were helpful in enabling optimal decision-making. Five attributes were detrimental to optimal decision-making. Two attributes had some aspects or sub-categories that were helpful, and other aspects that were detrimental to making optimal decisions. Table 3 presents the Generative AI attributes mentioned in the selected articles by category, subcategory, type, and frequency of attribute.

**Table 3:** Category, subcategory, type, and frequency of Generative AI attributes mentioned in the articles included.

ID	Attribute category	Frequency of attribute category	Attribute sub-category (and frequency)	Type of attribute
1.	Learning	21	+ Deep learning (6).	Helpful (+).
			+ Few-shot learning (2).	
			+ Learning (2).	
			+ Reinforcement learning from human feedback (RLHF) (2).	
			+ Zero-shot learning (2).	
			+ Advanced learning (1).	
			+ In-context learning (1).	
			+ Learning from specific inputs (1).	
			+ Learning structures from data (1).	
			+ Learning without explicit instruction (1).	
			+ Machine learning (1).	
			+ Unsupervised learning (1).	
2.	Understanding	18	+ Contextual understanding (10).	Some aspects are helpful, others detrimental (±).
			+ Understanding natural language (5).	
			+ Understanding trends from data (1).	
			- Inadequate or incorrect understanding (1).	
			- Superficial understanding of content and context (1).	
3.	Bias	14	- Algorithmic bias (3).	Detrimental (-).
			- Bias (3).	
			- Data bias (3).	
			- Amplification of bias (1).	
			- Gender bias (1).	
			- Racial bias (1).	
			- Response bias (1).	
			- Weighted towards socially desirable responses (1).	
4.	Pattern recognition	8	+ Pattern recognition (6).	Helpful (+).
			+ Identifying and understanding relationships (2).	
5.	Analysis	7	+ Analysis (2).	Helpful (+).
			+ Contextual analysis (2).	
			+ Analysing and drawing inferences from patterns (1).	
			+ Analysing the structure and meaning of text (1).	
			+ Analysis of complexity (1).	
6.	Ethical considerations	6	+ Influenced by the ethical guidelines of organisations like the WHO (1).	Some aspects are helpful, others detrimental (±).
			- Lack of consideration for ethics (3).	
			- Lack of consideration for human safety (1).	
			- Ethical questions in privacy, security, and reliability (1).	
7.	Lack of privacy	5	- Lack of consideration of privacy (5).	Detrimental (-).
8.	Knowledge	4	+ General knowledge (1).	Helpful (+).
			+ Knowledge (1).	
			+ Knowledge synthesis (1).	
			+ Specific domain knowledge (1).	
9.	Memory	4	+ Information retrieval (3).	Helpful (+).
			+ Memory (1).	
10.	Adaptability	4	+ Adaptability (4).	Helpful (+).
11.	Creativity	4	+ Creativity (3).	Helpful (+).
			+ Imagination (1).	
12.	Logic	3	+ Logic (2).	Helpful (+).
			+ Sequential processing (1).	
13.	Thinking	3	+ Human-like thinking (1).	Helpful (+).
			+ Structured thinking (1).	
			+ Thinking (1).	
14.	Cognition	2	+ Human-like behavioural patterns (1).	Helpful (+).
			+ Human-like cognitive abilities (1).	
15.	Reasoning	2	+ Reasoning (1).	Helpful (+).
			+ Step-by-step reasoning (1).	
16.	Ambiguity	1	- Ambiguity (1).	Detrimental (-).
17.	Innovation	1	+ Innovation (1).	Helpful (+).
18.	Attention	1	+ Attention (1).	Helpful (+).
19.	Lack of empathy	1	- Lack of empathy (1).	Detrimental (-).
20.	Hallucinations	1	- Hallucinations (1).	Detrimental (-).

Table 4 provides a summary of the articles included and lists the Generative AI attributes mentioned in each article.

**Table 4:** Summary of articles included and attributes of Generative AI mentioned in each article.

First author and year of publication	Title	Source	Summary	Generative AI attributes in the article
Davies et al. 2024	ChatGPT sits the DFPH exam: large language model performance and potential to support public health learning	ProQuest	This paper examines the possibility, and risk, of LLMs like ChatGPT creating infodemics by generating vast amounts of plausible-sounding but incorrect information that will have a negative impact on public health information. This paper evaluates ChatGPT in the context of the Faculty of Public Health's Diplomat exam (DFPH).	Deep learning and contextual understanding, such as the learning required to pass professional examinations, Hallucination of facts, Structured thinking (inferred from its high degree of accuracy on questions related to research methodology), Inadequate or incorrect understanding of context (inferred from its poorer performance on questions related to scenario-based questions on public health)
Miao et al. 2024	Chain of Thought Utilization in Large Language Models and Application in Nephrology	ProQuest	The context of this paper is nephrology, but it goes well beyond the clinical sphere. Apart from the use of AI in the clinical context (nephrology), it provides a high-level view of attributes that influence AI decision-making in healthcare, with detailed "chain of thought prompting" flow charts. It also deals with ethical concerns about AI in healthcare, data safety, the sensitive nature of medical data, and the ethical responsibility in healthcare decision-making.	Step-by-step reasoning (inferred from chain-of-thought prompting). Logic and sequential processing: The AI model processes information in a sequential manner, considering one aspect of the problem at a time, similar to how a human would logically break down a complex problem. Contextual understanding: The AI model can incorporate contextual variables, such as patient history, social determinants of health, or recent changes in medical guidelines, into its decision-making process--this helps the model weigh different factors and provide contextually appropriate recommendations or solutions
Sezgin 2023	Artificial intelligence in healthcare: Complementing, not replacing, doctors and healthcare providers	Google Scholar	This paper explores the role of AI, including Generative AI, in healthcare, and emphasises that AI is meant to complement, not replace, doctors, healthcare providers, and healthcare organisations. It highlights the importance of human-AI collaboration in healthcare organisations and the potential for AI to enhance healthcare outcomes.	Understanding natural language, Creation Innovation Human-like cognitive abilities Knowledge: General knowledge Knowledge of specific domains such as medicine Learning and improving from human feedback, Ability to analyse complexity, Adaptability
Cheng et al. 2023	WHO declares end of COVID-19 global health emergency: lessons and recommendations from the perspective of ChatGPT/GPT-4	Google Scholar	This paper is about the pandemic and ChatGPT's ability to analyse questions and provide answers and recommendations about COVID-19, such as spread, symptoms, diagnosis, treatment, vaccines, and pandemic management. AI, including ChatGPT/GPT-4, has the potential to safeguard public health and safety, by assisting in the study of spread routes, spread processes, and the epidemic laws of infectious diseases.	Machine learning, Deep learning, Contextual understanding and analysis
Deiana et al. 2023	Artificial intelligence and public health: evaluating ChatGPT responses to vaccination myths and misconceptions	Google Scholar	This paper explores ChatGPT in the context of public health. It is an evaluation of the ChatGPT's responses to vaccination myths and misconceptions.	Deep learning, Understanding natural language, Attention Imagination Thought Memory Interprets and perceives concepts, Bias Lack of consideration of privacy, Lacks a moral compass (lack of consideration of ethics)
Korzyński et al. 2023	Artificial intelligence prompt engineering as a new digital competence: Analysis of Generative AI technologies such as ChatGPT	ProQuest	This study reveals the profound implications of AI prompt engineering in healthcare.	Unsupervised learning, Pattern recognition, In-context learning via prompting, Zero-shot learning, Few-shot learning, Reasoning, Hallucinations, Response bias
Lorenz et al. 2023	Initial policy considerations for Generative Artificial Intelligence	Google Scholar	Initial policy considerations for Generative AI, discussing its transformative potential and the challenges it poses in healthcare and other sectors.	Few-shot learning: Learning content with only a few examples or training instances. Amplifying bias found in the data
Mannuru et al. 2023	Artificial intelligence in developing countries: The impact of Generative Artificial Intelligence (AI) technologies for development	Google Scholar	This paper explores the potential impact of Generative AI technologies on developing countries, considering both positive and negative effects across healthcare and other domains.	Creativity and innovation, Advanced learning, Pattern recognition, Understanding natural language, Contextual understanding, Understanding trends from the data, Adaptability: Generative AI can adapt to different contexts and adjust its decision-making based on the input it receives and the desired outcome
Meskó 2023	The ChatGPT (Generative Artificial Intelligence) Revolution Has Made Artificial Intelligence Approachable for Medical Professionals	Google Scholar	This paper explores the impact of the ChatGPT revolution in making artificial intelligence more accessible for medical professionals and explores the various AI tools and services that have become available in the healthcare industry.	Pattern recognition, Learning Adaptability
Meskó 2023	Prompt engineering as an important emerging skill for medical professionals: tutorial	Google Scholar	This paper explores the importance of prompt engineering as an emerging skill for medical professionals in the context of large language models (LLMs) and artificial intelligence (AI) in healthcare. It also explores how Generative AI prompt engineering can improve various healthcare domains, including public health initiatives, and the limitations and risks of Generative AI.	Learning from specific inputs like prompt engineering to modify its decisions on “what information to select” and “what response to provide”
Meskó et al. 2023	The imperative for regulatory oversight of large language models (or Generative AI) in healthcare	ProQuest	This paper is a discussion on the need for regulatory oversight of LLMs in healthcare and the challenges and implications of implementing LLMs in medical settings.	Learning, Understanding natural language, Greater weighting given to socially desirable responses: LLMs can adapt their responses in real-time, based on user input and evolving contexts--this behaviour demands regulatory oversight
Miao et al. 2023	A future of smarter digital health empowered by Generative Pretrained Transformer	Google Scholar	GPT offers opportunities to improve or renovate digital health interventions and digital health-enabled care. Wearable AI-based devices and communication technologies like real-time augmented reality and streaming data platforms can integrate digital technology, medicine, behaviour, health care, and community living for the improvement of population and individual health. This highlights the importance of integrating digital health tools and AI-based devices to create a more intelligent digital health ecosystem.	Analysing data (collection of facts) to find information (facts in context) that is relevant to a given task, Human-like thinking, logic, knowledge, and behavioural patterns, Deep learning
Nova 2023	Generative AI in healthcare: advancements in electronic health records, facilitating medical languages, and personalised patient care	Google Scholar	This paper is about the application of Generative AI techniques in healthcare, specifically focusing on advancements in electronic health records, simplifying medical language, and providing personalised patient care.	Contextual understanding, Analysing the structure and meaning of the text, identifying key phrases and entities Identifying and understanding relationships between words and sentences, Understanding the context of conversations: identifying and categorising named entities within text, such as patient names, medical conditions, medications, and procedures
Ong et al. 2023	GPT Technology to Help Address Longstanding Barriers to Care in Free Medical Clinics	Scopus	The context of this paper is free clinics, but it also deals with health equity, and enhance comprehensive and holistic care in resource-limited settings. The authors comment on the use of ChatGPT in pre-clinic, peri-clinic, and post-clinic services, including patient transportation to free clinics, directing patients to convenient labs or image services, screening for discounts on medications, provide helpful health information, etc. Therefore, this paper is relevant to this review, because it is not only about the use of ChatGPT to help patients who attend free clinics, but also about public health and health equity in resource-poor settings.	Contextual understanding, Contextual analysis, Ambiguity Information retrieval
Oniani et al. 2023	From Military to Healthcare: Adopting and Expanding Ethical Principles for Generative Artificial Intelligence	Google Scholar	The paper explores the adoption and expansion of ethical principles for Generative AI in the healthcare industry, drawing parallels between the military and medical service.	Influenced by the principles and ethical guidelines set by various organisations, including the U.S. Department of Defense, NATO, and the World Health Organisation Bias Lack of consideration of human safety Lack of empathy
Ooi et al. 2023	The potential of Generative Artificial Intelligence across disciplines: Perspectives and future directions	Google Scholar	This paper is about the potential of Generative AI across disciplines including healthcare. In the context of healthcare, it explores Generative AI in patient care and service innovation, concerns about privacy, data ownership, bias, ethical and legal implications of using Generative AI in healthcare, and guidelines and regulations.	Learning without any explicit instructions (this is a part of machine learning), Adaptability (this is a part of machine learning), Pattern recognition Analysing and drawing inferences from patterns in data (this is a part of machine learning), Recognising and learning the structures in data Recognising relationships present in training data (neural networks), Data bias, Algorithmic bias, Lack of consideration for privacy
Parray et al. 2023	ChatGPT and global public health: applications, challenges, ethical considerations and mitigation strategies	Google Scholar	This paper explores the applications, challenges, and ethical considerations of using ChatGPT in global health research, electronic health records, etc.	Deep learning, Reinforcement learning from human feedback (RLHF), Contextual understanding and analysis Creativity Algorithmic bias, Data bias, Lack of consideration of ethics (lack of a moral compass), Lack of consideration for privacy
Patsakis and Lykousas 2023	Man vs the machine in the struggle for effective text anonymisation in the age of large language models	ProQuest	The challenges and importance of text anonymisation in the age of large language models, focusing on the balance between privacy protection and data utility. It also explores the challenges in the collection and use of personal data in healthcare, law, and research industries and raises ethical concerns around confidentiality and privacy.	Understand the structure and meaning of natural language text by tokenisation, part-of-speech tagging, named entity recognition, and syntactic parsing. Understanding context and input Information retrieval and memory: GPT-3 is trained on a large corpus of text data, including books, articles, and websites, with a primary source being the Common Crawl, a repository of web pages and documents. Reinforcement learning from human feedback (RLHF), which fine-tunes GPT to improve its performance on various text generation tasks. Understanding relationships and associations between different pieces of information. Pattern recognition (named entity recognition). Zero-shot learning
Rane et al. 2023	Contribution and performance of ChatGPT and other Large Language Models (LLM) for scientific and research advancements: a double-edged sword	Google Scholar	This paper explores the contribution and performance of ChatGPT and other LLMs in scientific and research advancements, in various fields, including public health.	Contextual understanding, Knowledge synthesis, Bias Lack of ethical considerations (lack of a moral compass), Lack of adequate consideration of privacy, Information retrieval (“memory”), Pattern recognition
Rodgers et al. 2023	Open Data and transparency in artificial intelligence and machine learning: A new era of research	ProQuest	The errors of AI in critical areas like healthcare, and the importance of open data, transparency, trust, and reliability.	Bias--racial bias and gender bias, Deep learning, Ethical questions in privacy, security and reliability, Superficial understanding of context, including legal and social implications
Wang et al. 2023	Ethical considerations of using ChatGPT in health care	Google Scholar	This paper is a discussion on various Generative AI attributes and ethical and legal considerations in the use of ChatGPT in healthcare. It explores legal responsibility, privacy issues, licencing, and regulation from government and from society, and the potential impact of Generative AI on the physician-patient relationship.	Algorithmic bias: Biased feature selection, model design, or decision rules, Data bias, based on bias embedded in the data, either due to biased sampling methods or human bias in data sources, Variable consideration, or lack of consideration, of privacy principles

Discussion

The articles included in this review indicate that Generative AI commands an impressive array of attributes that may contribute to optimal decision-making. Attributes that help Generative AI make optimal decisions are frequently mentioned, including learning, pattern recognition, analysis, knowledge, memory, adaptability, and creativity. However, attributes detrimental to making good decisions are also frequently mentioned, including bias, lack of privacy, lack of empathy, ambiguity, and hallucinations. In addition, attributes such as understanding and ethical considerations contain some aspects or sub-categories that are helpful and others that are detrimental to making appropriate decisions.

Learning

Learning is the most frequent attribute mentioned in the selected articles (n=21). Several types of learning are mentioned. Six articles mention deep learning, which simulates the human ability to make complex decisions [62-67]. Two articles discuss few-shot learning, an attribute that enables Generative AI to learn patterns in data after only a few examples of training [68,69]. Two articles discuss zero-shot learning, which allows AI to correctly identify and categorise concepts and objects that it has not explicitly encountered in training [68,70].

Parray et al. [66] and Patsakis and Lykousas [70] mention reinforcement learning from human feedback (RLHF). Parray et al. [66] state that this attribute enhances ChatGPT’s ability to generate human-like language, understand user intent, and maintain coherence in conversations, making ChatGPT increasingly useful in healthcare. Meskó [71] argues that learning from specific inputs like prompt engineering optimises decision-making in LLMs by enhancing their ability to select the most relevant information and provide the best response. Other attributes mentioned in the selected articles in relation to learning are advanced learning [72], machine learning [64], unsupervised learning and in-context learning [68], learning without explicit instruction and learning structures from data [73].

Meskó [74] states that continuous learning is one of the attributes that has made AI tools approachable and accessible to healthcare professionals. Meskó and Topol [75] discuss AI learning, arguing that this attribute's scale, capability, versatility, and impact in LLMs differ significantly from the specialised learning capabilities of earlier neural networks. Therefore, regulatory frameworks for LLMs must be adaptable, so that oversight can be specifically tailored to specialist domains like healthcare [75].

Understanding

Understanding (n=18) is another attribute that is frequently mentioned. Different types of understanding are mentioned in the articles included in this review. Ten articles discuss contextual understanding as an important attribute of Generative AI [62,63,65, 66,70,72,76-79]. Deiana et al. [64] argue that contextual understanding, deep learning, and machine learning have allowed ChatGPT to advance the study of public health, enhancing the understanding of the routes, processes, and epidemic laws that govern the spread of infectious diseases.

Five articles mention that Generative AI understands natural language [64,70,72,75,80]. Deiana et al. [64] found that while ChatGPT’s responses were easy to understand and reasonably (85.4%) accurate, they also misinterpreted questions and provided misleading answers. The paid version of ChatGPT provided more accurate answers. This raises further ethical concerns, considering the existing social divide on access to healthcare information [64].

Generative AI understands trends from data, which allows it to predict health outcomes [72]. However, it has a superficial understanding of content and context [67], and may have an inadequate or incorrect understanding of context [62]. Mannuru et al. [72] highlight the contextual limitations of Generative AI, for instance, it may overlook the social contexts of healthcare. Rodgers et al. [67] state that Generative AI takes all data at face value unless taught otherwise, which raises the ethical question of its reliability in healthcare, where AI errors can be fatal.

Pattern Recognition

Pattern recognition is another frequently mentioned attribute (n=8). Generative AI is trained on large amounts of labelled data to learn patterns and correlations and identify and understand relationships within the data [77]. It identifies patterns in the data [68], identifies relationships between words and phrases [72], and generates new data through the process of recognising patterns [74]. In fact, its ability to synthesise new data--its generative nature--is based on recognising patterns [73]. Pattern recognition is one of the attributes of Generative AI that can be used to inform public health interventions and initiatives [79].

Analysis

Analysis is a Generative AI attribute [63] that can be applied to large amounts of data to assist in understanding public health [66]. For instance, Generative AI can analyse data to help understand the risk factors for various diseases [66]. Generative AIs like ChatGPT are significantly better than search engines in analysing and responding to a patient's input, because they are able to tailor answers to the patient's specific preference or circumstance [78]. In fact, ChatGPT can analyse data better than many entry-level and mid-level human professionals [65]. However, for tasks that require a more subtle grasp of knowledge in specific domains, Generative AI’s analysis lacks a deeper understanding of word meanings, which can generate responses that not only lack discernment but also digress from the topic [73]. Sezgin [80] recommends that the analytical capabilities of Generative AI should be deployed as a complement to the cognitive strengths of healthcare providers, a human-in-the-loop (HITL) approach that ensures quality and safety in healthcare.

Ethical Considerations

Deiana et al. [64], Parray et al. [66], and Rane et al. [79] discuss Generative AI’s lack of consideration for ethics. Rodgers et al. [67] raise ethical questions about the privacy, security, and reliability of Generative AI, while Oniani et al. [81] state that Generative AI lacks consideration for human safety. However, Generative AI with appropriate programming is influenced by the ethical guidelines of organisations like the World Health Organisation (WHO) [81].

Wang et al. [82] discuss the ethical guardrails required for the use of ChatGPT in healthcare. The authors discuss the need to ensure legal, humanistic, algorithmic, and information ethics. Legal ethics include legal responsibility, appropriate provisions for privacy, and licencing and regulation; humanistic ethics consist of humanistic care, integrity, and measures that safeguard the physician-patient relationship; algorithmic ethics include algorithmic responsibility, transparency, explainability, evaluation and validation; and information ethics include validating information and validating the effectiveness of ChatGPT in healthcare [82]. Sezgin [80] states that advances in Generative AI promise to create a positive paradigm shift by enhancing and supporting the skills of healthcare professionals and providers. However, the author underlines the importance of appropriate controls and guidance for Generative AI in healthcare [80].

Generative AI can be biased [64,79,81] and can also amplify bias that exists in the data [69]. Furthermore, it is prone to data bias, based on errors or prejudice in the data, and algorithmic bias, based on limited input, skewed data, and unfair, exclusionary algorithms [66,73,82]. Other types of Generative AI bias include racial bias and gender bias [67]. Korzynski et al. [68] equate Generative AI with a stochastic parrot that mimics and reproduces text without a deep understanding of context or content. This response bias can result in prejudiced, irrelevant, or inappropriate outputs. LLMs have a propensity for socially desirable responses, adapting their response based on human input and evolving context--a behaviour that has ethical implications and requires regulatory oversight [75].

Five articles discuss Generative AI’s lack of consideration for privacy [64,66,73,79,82]. While there may be strict privacy regulations and legal provisions for AI technologies specifically designed for healthcare, these regulations do not apply to Generative AI because it was designed for a general audience, not healthcare professionals [74]. Patsakis and Lykousas [70] explore the challenges of text anonymisation in the age of large language models, which also raises ethical concerns about the confidentiality of patient data.

Generative AI like ChatGPT lacks empathy, highlighting the need to incorporate the principles of empathy into the Generative AI used in healthcare [81]. Ong et al. [78] state that prompt engineering techniques are important because they can reduce ChatGPT’s ambiguity, influence its style and tone, and enable it to retrieve specific information. Davies et al. [62] examine AI’s propensity for hallucinations, and the risk of LLMs like ChatGPT creating public health infodemics. As LLMs are mainly owned and developed by private corporations, the authors stress the critical importance of independently verifying AI capabilities, and AI’s capacity for both good and bad outcomes [62].

Other Human-Like Attributes

Generative AI has human-like knowledge [65,80]. Sezgin [80] states that it has general knowledge as well as the knowledge of specific domains such as medicine. Rane et al. [79] state that knowledge synthesis is a Generative AI attribute that affords the opportunity to advance numerous disciplines, including public health.

Ong et al. [78], Patsakis and Lykousas [70], and Rane et al. [79] mention memory and information retrieval as attributes of Generative AI. Patsakis and Lykousas [70] state that GPT-3, the third generation of the Generative Pre-trained Transformer, can retrieve information from a large corpus of books, articles, websites, and many other sources of text data; its primary source being the repository of documents and web pages known as the Common Crawl. Four articles comment on Generative AI’s adaptability [72-74,80]. Mannuru et al. [72] state that Generative AI can adapt to different contexts and adjust its decision-making based on the input and the desired outcome [80]. Notes that this adaptability improves with human feedback, enabling Generative AI to handle greater complexity.

Generative AI can be creative [66,72,80]. It has imagination [64] and can innovate [72,80]. It can think [76] and is capable of human-like thinking [65] and structured thinking [62]. It has cognitive abilities [73,80], even human-like cognition [80], and can pay attention [64].

Miao et al. [65] state that Generative AI has human-like logic. Miao et al. [76] comment on its logic, sequential processing, and step-by-step reasoning, which give it the ability to consider one aspect of the problem at a time and logically break down complex problems as a human would. Miao et al. [76] also explore chain-of-thought prompting, a technique that can enhance the ability of LLMs to manage complexity and make contextual judgments. However, they argue that the ethical implication of using such techniques is a critical gap in the current discourse on AI in healthcare.

The methods used in this review are as transparent and rigorous as possible. It adopts the guidelines for rapid reviews described by Dobbins in 2017 [56]. The review has limitations that may well have resulted in some relevant articles being missed. The review only includes peer-reviewed articles published in English in 2023 and early 2024. Searching additional systems and databases may have yielded more results. Therefore, ongoing and further research are necessary.

## Conclusions

This rapid review has demonstrated that Generative AI possesses significant capabilities in learning, pattern recognition, and analysis. However, its decision-making in complex health services is undermined by important limitations in understanding, ethical considerations, and bias mitigation. Generative AI has no concept of conscience and no moral compass. Unless specifically programmed, Generative AI seems to lack consideration of ethical principles, human safety, and privacy. Furthermore, it is not transparent and can neither take responsibility nor be held accountable for its output or outcomes. It also lacks empathy and has a propensity for bias and hallucinating facts. These results suggest that more work needs to be done before Generative AI can be applied to complex health services.

As humans interact with increasingly intelligent machines, many ethical questions arise about the impact of such interaction. While Generative AI brings the promise of advanced automation on a scale never before seen or imagined, there is a significant risk that it can be manipulated by human beings. It could also be argued that humans have now entered the age of Generative AI--an era not defined by the nature of artificial intelligence, but by the human condition. It is now more urgent than ever to examine how artificial intelligence makes decisions. It is vital to ensure a principled and ethical approach to AI design, engineering, and use that is underpinned by human values. Which values do humanity want to uphold? Will we find a way to program an AI with kindness and empathy. What are we building and who decides the optimal recipe?
